# Do critical illness survivors with multimorbidity need a different model of care?

**DOI:** 10.1186/s13054-023-04770-6

**Published:** 2023-12-08

**Authors:** Jonathan Stewart, Judy Bradley, Susan Smith, Joanne McPeake, Timothy Walsh, Kimberley Haines, Nina Leggett, Nigel Hart, Danny McAuley

**Affiliations:** 1 Centre for Experimental Medicine, Wellcome-Wolfson Institute for Experimental Medicine, Queen’s University Belfast, Belfast, Northern Ireland; 2 Centre for Medical Education, Queen’s University Belfast, Belfast, Northern Ireland; 3 Department of Public Health and Primary Care, Trinity College Dublin, Dublin 2, Ireland; 4 The Healthcare Improvement Studies Institute, Department of Public Health and Primary Care, University of Cambridge, Cambridge, UK; 5 Usher Institute, University of Edinburgh, Edinburgh, Scotland, UK; 6 Department of Critical Care, Melbourne Medical School, University of Melbourne, Melbourne, Australia

**Keywords:** Critical illness, Multimorbidity, Transitions of care

## Abstract

There is currently a lack of evidence on the optimal strategy to support patient recovery after critical illness. Previous research has largely focussed on rehabilitation interventions which aimed to address physical, psychological, and cognitive functional sequelae, the majority of which have failed to demonstrate benefit for the selected outcomes in clinical trials. It is increasingly recognised that a person’s existing health status, and in particular multimorbidity (usually defined as two or more medical conditions) and frailty, are strongly associated with their long-term outcomes after critical illness. Recent evidence indicates the existence of a distinct subgroup of critical illness survivors with multimorbidity and high healthcare utilisation, whose prior health trajectory is a better predictor of long-term outcomes than the severity of their acute illness. This review examines the complex relationships between multimorbidity and patient outcomes after critical illness, which are likely mediated by a range of factors including the number, severity, and modifiability of a person’s medical conditions, as well as related factors including treatment burden, functional status, healthcare delivery, and social support. We explore potential strategies to optimise patient recovery after critical illness in the presence of multimorbidity. A comprehensive and individualized approach is likely necessary including close coordination among healthcare providers, medication reconciliation and management, and addressing the physical, psychological, and social aspects of recovery. Providing patient-centred care that proactively identifies critical illness survivors with multimorbidity and accounts for their unique challenges and needs is likely crucial to facilitate recovery and improve outcomes.

## Introduction

Critical illness is not a single condition, but instead captures the experience of a heterogeneous group of patients whose commonality is that their illness is so severe that it requires advanced organ support within an Intensive Care Unit (ICU) [1]. Internationally between 75 and 90% of people admitted to ICU with a critical illness survive to hospital discharge [2–5]. Finding strategies to support ICU survivors when they return home is considered a top ICU research priority by patients, carers and health professionals [6]. Evidence generated over the last two decades has shown that critical illness survivors often experience long-term physical, psychological, and cognitive sequelae as a direct result of the acute illness, commonly known as post-intensive care syndrome (PICS) [7]. The majority of existing post-ICU models of care and clinical guidelines have focussed on mitigating these functional complications. However, trials focussed on rehabilitation interventions to mitigate functional impairments after critical illness have failed to demonstrate benefit in long term outcomes [8].

**Fig. 1:**
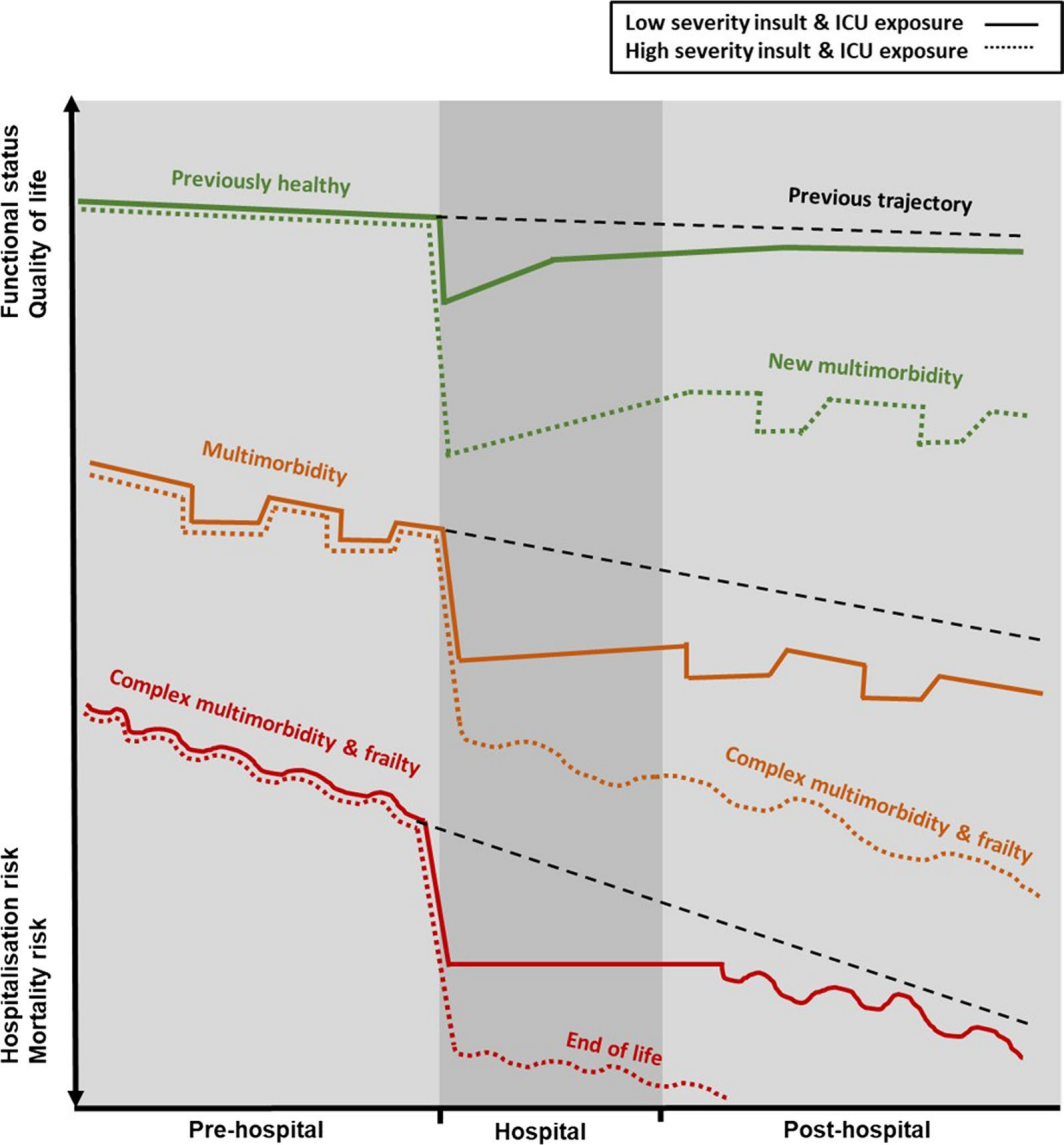
Distinct recovery trajectories before, during and after critical illness. Recovery trajectories vary depending on pre-illness health status. Black hatched lines indicate health trajectories that would have occurred if the individual had not experienced a critical illness. Previously healthy individuals who experience a less severe illness are more likely to recover to their pre-illness functional and health status compared to previously other previously healthy individuals who experience a more severe illness. For individuals with multimorbidity, particularly if they are also frail, their baseline pre-illness functional status and trajectory is likely to be worse, their recovery is likely to be slower, and they are less likely to recover to their pre-illness health and functional status. (Adapted from Iwashyna [ 12 ] and Latronico [ 15 ])

There is increasing recognition that a person’s pre-existing health status, and in particular the presence of multiple long-term conditions, or multimorbidity, is a key determinant of long-term outcome after ICU [9–11]. Multimorbidity is often defined as the co-existence of at least two chronic conditions in an individual [12]. This definition has come under criticism for being too simplistic as it includes combinations of well controlled or relatively mild conditions which may not meaningfully impact patients (e.g. hypertension and well controlled diabetes). There have been calls for alternative definitions which consider not only the number of conditions, but also their severity and impact, and where the condition combinations are more likely to significantly impact a person’s daily life and risk of deterioration (e.g. heart failure, depression and back pain). Despite the criticisms, recent evidence indicates that critical illness survivors with multimorbidity have significantly worse recovery trajectories and outcomes compared to previously ‘healthy’ patients [13–18] (Fig. 1). Iwashyna (2012) hypothesised that three distinct critical recovery trajectories exist, (1) the ‘’big hit’’ (characterized by acute functional decline followed by recovery), (2) the ‘’slow burn’’ (characterised by constant decline over time); and (3) “relapsing recurrences” (characterised by repeated acute exacerbations and partial recoveries) [14]. Building on this work, Latronico et al. (2017) hypothesised that the trajectories of critical illness survivors can be further divided based on the patient’s pre-illness health status including pre-existing medical conditions [15]. Recent studies investigating critical illness trajectories have provided further evidence that pre-existing multimorbidity and high healthcare utilisation are better predictors of hospital readmission and mortality than severity of the acute illness [19, 20].

These studies indicate that critical illness survivors with multimorbidity represent a distinct recovery subtype. Unlike previously ‘healthy’ patients whose recovery trajectory is predominately impacted by the severity of the acute ‘‘big hit’’ of critical illness, for patients with multimorbidity the impact of the acute illness may be overwhelmed by pre-illness factors [13]. This raises the questions of what factors predispose critical illness survivors with multimorbidity to worse outcomes, and which of these factors are potentially modifiable and amenable to treatment.

In this narrative review we explore two main questions: (1) Why is multimorbidity associated with worse outcomes after critical illness? and (2) How can the care of critical illness survivors with multimorbidity be enhanced to improve outcomes?

## Why is multimorbidity associated with worse outcomes after critical illness?

**Fig. 2:**
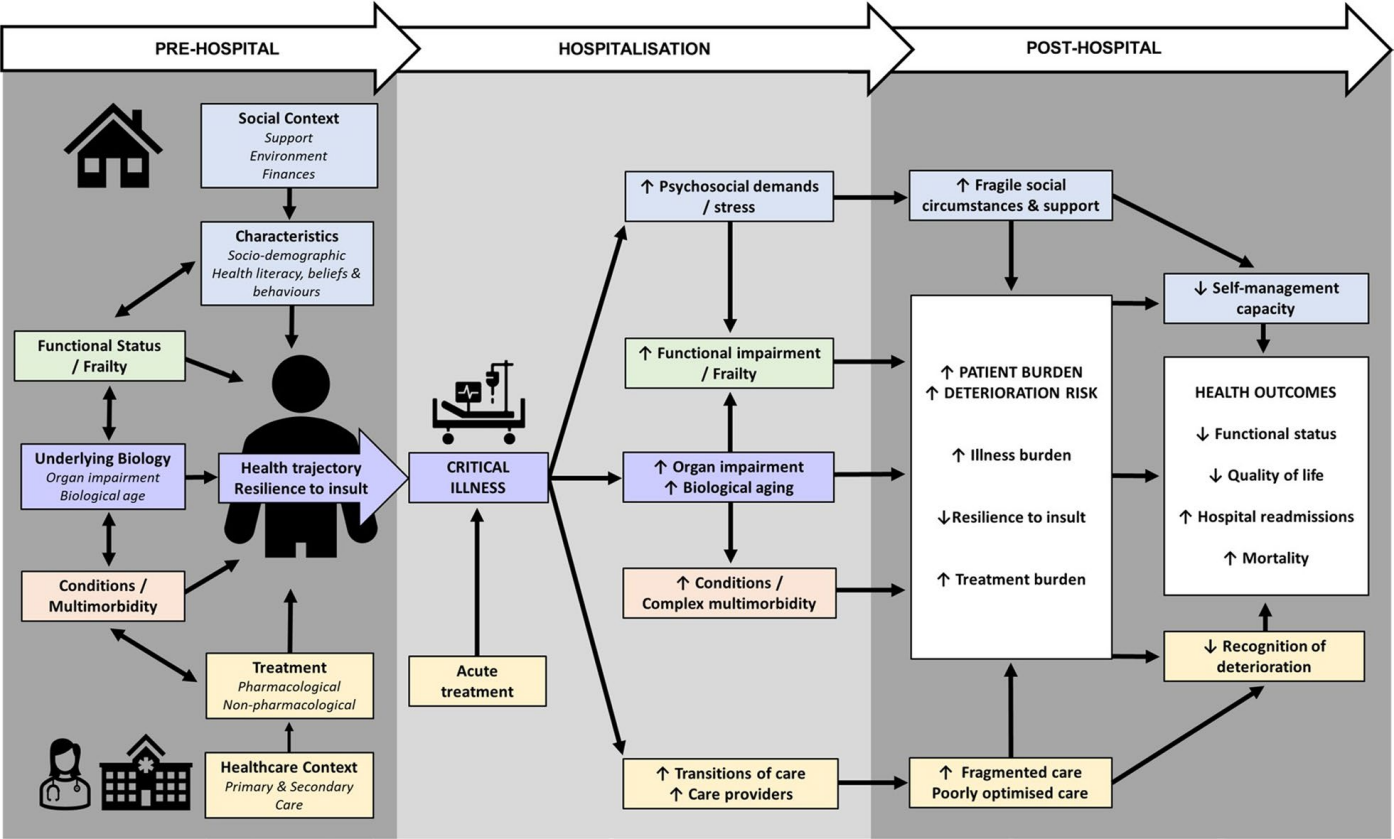
Factors which likely play an important role in mediating the relationship between multimorbidity and outcomes prior, during and after critical illness. Including underlying biology and pathophysiology, condition and multimorbid effects, functional impairment and frailty, social context, treatment burden and healthcare context. Resilience is defined as ability to cope with and recover from an acute stressor

The relationship between multimorbidity and outcomes amongst critical illness survivors is complex. Building on previous research from areas including critical care and primary care we have developed a conceptual model through which we explore six closely related domains which likely play an important role in mediating the relationship between multimorbidity and outcomes prior to, during and after critical illness (Fig. 2);

- Underlying biology and pathophysiology
- Condition and multimorbid effects
- Functional impairment and frailty
- Social context
- Treatment burden and risk
- Healthcare context

### Underlying biology and pathophysiology

The biological and pathophysiological mechanisms underlying the associations between multimorbidity and outcomes after critical illness are further complicated by the heterogeneity of the critically ill population. However, there are some common factors which are likely involved.

#### Organ impairment and resilience to insult

Critical illness is defined by the presence of organ impairment which is severe enough to require advanced support in an ICU. Chronic conditions are commonly the result of organ dysfunction, for example reduced pulmonary function in chronic obstructive pulmonary disease (COPD), cardiac function in chronic heart failure, or renal function in chronic renal failure. Therefore, patients with multimorbidity usually have multiple organs with impaired function. The resilience of these impaired organs (defined as their ability to cope with and recover from an acute stressor) may be reduced, and a relatively minor illness could result in the requirement for organ support. Organ impairment also manifests as functional impairments (e.g. physical weakness, psychological impairment and cognitive impairment) which have complex bidirectional relationships with multimorbidity and will be explored further below [21].

#### Biological Ageing

Age is the most important risk factor for multimorbidity [22]. People aged over 65 years represent an increasing proportion of the ICU population [23], and age is consistently identified as a predictor of worse outcomes after ICU. However, age has a complex relationship with the heath trajectory of critical illness survivors. While at a population level, pre-illness factors such as older age appear better predictors of hospital readmission, for the subgroup of patients without pre-existing conditions acute illness factors appear to be better predictors of rehospitalisation [19, 24]. This may be partly related to the distinction between chronological and biological age. While chronological Ageing refers to the amount of time a person has existed, biological Ageing refers to the time dependent accumulation of cellular damage [25, 26]. Many chronic conditions are increasingly recognised as the manifestation of accelerated hallmarks of biological Ageing including diabetes and cardiovascular disorders [25, 27–30]. Biologically older people, with their associated multiple chronic conditions and functional impairments, are more vulnerable to deterioration prior to, during and after critical illness.

Critical illnesses may also drive biological Ageing (Fig. 2). Chronic inflammation is a hallmark of biological Ageing [26, 27]. Critical illness syndromes, including sepsis, are potent drivers of inflammation [31]. Persistent inflammation following critical illness is associated with the development of chronic conditions including new renal and cardiovascular disorders [32–34] and new functional deficits [35].

Another important ‘pillar of Ageing’ is adaption to stress [25, 27, 30, 36]. Allostatic load represents the physiological consequences of heightened neuroendocrine response to chronic stress [37]. It has been associated with development of a range of health conditions including diabetes mellitus, musculoskeletal disorders, and cancer [28]. Critical illness may represent a stress-related decompensation syndrome leading to organ failure [38, 39]. Ongoing stress following hospital discharge may drive biological Ageing and compromise recovery [39].

### Specific conditions and multimorbidity clusters

Interventions to optimise the care of patients with multimorbidity are usually agnostic to specific conditions, and instead focus on complications which result from having multiple diseases simultaneously, including high illness burden, high treatment burden, polypharmacy related issues, self-management challenges, and poorly coordinated care [29]. While the focus of this review is the cumulative impact of multiple chronic conditions on health trajectory during and after critical illness, specific medical conditions have important effects prior to, during and after the acute illness. Over the last two decades, multiple observational studies have identified associations between specific pre-existing conditions and worse short and long-term patient outcomes after critical illness including poorer quality of life [40], hospital readmission [19, 20, 24, 41] and mortality [9, 10, 20, 24, 42, 43]. Certain pre-existing conditions such as chronic liver disease, chronic renal disease, and malignancy consistently have strong associations with long-term mortality risk [9, 10, 42, 44]. This may relate to their severity or difficultly modifying the trajectory of these conditions. However, the associations should be interpreted with caution, and do not necessarily imply causation. For example, decisions regarding escalation of care and hospital readmission may be impacted by the presence of certain severe or unmodifiable conditions. Nevertheless, identifying potentially modifiable patient risk and burden related to conditions and optimising care may improve outcomes.

As well as physical illnesses, mental illnesses (including depression and anxiety) are common amongst critical illness survivors and associated with worse outcomes [45–47]. The associations between multimorbidity, mental illness and worse health outcomes are well established in the wider population outside ICU. Patients with multimorbidity in the general population whose condition profile includes depression, pain or psychoactive substance misuse have significantly higher healthcare utilisation and mortality rates compared to patients without these conditions [48].

Patients also commonly develop new conditions following critical illness. In a cohort of sepsis and ARDS survivors, Jouan et al. (2019) found higher rates of renal, respiratory, and cardiac conditions in the post-ICU period, demonstrating the role of critical illness as a driver of multimorbidity [49]. Critical illness survivors may also have undiagnosed conditions. For example, patients admitted to the ICU with acute hypercapnic respiratory failure have high rates of COPD, obstructive sleep apnoea (OSA) and heart failure [50]. These often-undiagnosed conditions are associated with higher rates of hospital readmission and mortality and are a potential target for future interventions.

As well as their individual effects, multiple conditions can lead to interactions and synergistic effects that increase illness burden and risk of deterioration. The combinations of conditions patients experience after critical illness are unlikely to occur randomly. Conditions often cluster together and share pathological mechanisms [51]. Clusters of pre-existing conditions have been identified, through machine learning techniques, which are associated with poor outcomes in mixed [52] and COVID-19 [53] ICU populations. These include condition clusters around renal failure and cardiovascular disease with high associated mortality [32, 52, 53].

### Functional impairment and frailty

There are strong bidirectional links between multimorbidity and functional impairment [21]. The functional sequelae of critical illness, or PICS, likely share some biological and pathophysiological mechanisms with new and existing conditions including increased organ impairment (e.g. skeletal muscle or brain) and biological Ageing (e.g. inflammation) (Fig. 2). The overlaps may amplify illness burden and risk of deterioration, and negatively impact the ability of patients to self-manage and access care.

There has been a particular research focus on identifying interventions to address the physical functional impairment aspect of PICS (commonly referred to as ICU acquired muscle weakness), most trials failing to demonstrate patient benefit [8, 54, 55]. However, a recent meta-analysis using individual patient data from four of these trials found critical illness survivors with multimorbidity may be more likely to respond to physical rehabilitation interventions than patients without pre-existing medical conditions [56]. This provides further evidence for strong bidirectional links between multimorbidity and functional impairment and suggests a targeted approach to physical rehabilitation based on the pre-existing multimorbidity status of critically ill patients may be beneficial.

At the extreme end of functional impairment is frailty, which is characterised by significantly decreased physical, psychological, and cognitive reserves and increased vulnerability to an external stressor event [57]. Pre-existing frailty is associated with worse outcomes during and after ICU admission [58–62]. However, recent research indicated that a significant proportion of frailty seen amongst critical illness survivors after hospital discharge is newly acquired, even amongst patients of younger chronological age [63, 64], providing further evidence that critical illness is a driver of biological Ageing. Multimorbidity is related to frailty, however while most frail individuals have multimorbidity, a relatively small proportion of people with multimorbidity are frail [65, 66]. Therefore, patients with pre-existing multimorbidity and frailty represent a particularly vulnerable cohort of ICU survivors, likely driven by the limited potential to modify the already downward trajectory of these patients (Fig. 1).

### Social context

The physical, psychological and social wellbeing of critical illness survivors are closely correlated [67]. Critical illness survivors from areas of higher socio-economic deprivation have higher rates of multimorbidity and polypharmacy [68], lower quality of life and higher mortality following hospital discharge [69, 70]. The links between critical illness, multimorbidity, and socio-economic deprivation are complex, however may be partly explained by accelerated biological Ageing. People growing up under conditions of socioeconomic disadvantage exhibit a faster pace of biological Ageing [71–73], experience higher stress and allostatic load [74], and are more likely to develop multimorbidity at a younger chronological age, particularly multimorbidity that includes mental health disorders [22]. This accelerated biological Ageing and associated multimorbidity could predispose to worse outcomes during and after critical illness.

Critical illness also results in new social and financial problems for patients. Financial problems related to employment disruption are common following critical illness and associated with worse health-related quality of life and psychological function [75–80]. Patients exposed to ICU are also more likely to experience social isolation than other hospitalised patients [67]. Absence of adequate social support is associated with worse outcomes after critical illness including disability and mortality [81], which may be partly explained by difficulty self-manAgeing multiple new and existing medical and functional issues. When support is available, it is commonly provided by informal carers such as family members, leading to significant carer burden [82].

### Treatment burden and risk

Multimorbidity is usually associated with high treatment burden (defined as workload demands on patients to manage treatment and healthcare recommendations) [83]. One of the major drivers of high treatment burden is the cumulative implementation of multiple single condition guidelines without consideration of the overall patient impact [84–86]. Polypharmacy (often defined as five or more regular medications) is common in the post-ICU population (> 30%) and is an independent predictor of hospital readmission, even after adjustment for pre-existing medical conditions [68]. Medication related issues (e.g., prescribing and reconciliation errors) are common amongst critical illness survivors following hospital discharge (> 55%) with a significant proportion related to analgesic or psychiatric medications [87, 88].

Treatment burden is not only driven by medications. Critical illness survivors with multimorbidity may experience fragmented care with multiple appointments with various healthcare providers following hospital discharge [89–91]. There is also a significant educational and information burden associated with self-management of multiple new and existing conditions. The combination of high illness burden, high treatment burden, fragile social support and fragmented care significantly reduces the capacity of patients to self-manage their care, including for chronic conditions [83, 92, 93]. Critical illness survivors have identified difficulty self-manAgeing multiple conditions as a key driver of hospital readmissions [82].

### Health and social care context

Care for patients with multimorbidity is commonly fragmented and uncoordinated, as healthcare systems are generally designed around single conditions, rather than provision of holistic and comprehensive patient centred care [29]. Compared to other hospitalised patients, critical illness survivors experience additional care transitions to and from the ICU [89, 94]. An important potential driver of fragmented care for critical illness survivors with multimorbidity is the lack of clarity on which professional groups are responsible for arranging and coordinating the various aspects of follow-up following hospital discharge, including for new and existing conditions [91]. The resultant fragmented and poorly coordinated care increases the likelihood that deterioration will go unrecognised by care providers.

## How can we enhance the care of critical illness survivors with multimorbidity?

Critical illness survivors with multimorbidity are uniquely vulnerable to deterioration compared to other ICU and hospitalised patients. Current guidance on provision of ICU follow-up places emphasis on addressing functional sequelae and symptoms which can be directly attributed to the critical illness and ICU exposure [95, 96]. For patients with multimorbidity, their care also needs to consider their pre-existing health status and related factors. Much of the research into the optimal model of care for patients with multimorbidity comes from primary care, however the majority of trials have failed to demonstrate benefit [97]. A recent review article summarised factors which likely need to be considered when for providing care for patients with multimorbidity to inform future research [29];

- Optimisation of conditions.
- Optimisation of related factors including functional impairment, frailty, and social circumstances.
- Treatment burden, self-management support and care coordination.
- Personalised care based on patient priorities and preferences.
- Family and informal carer orientation.

**Fig. 3:**
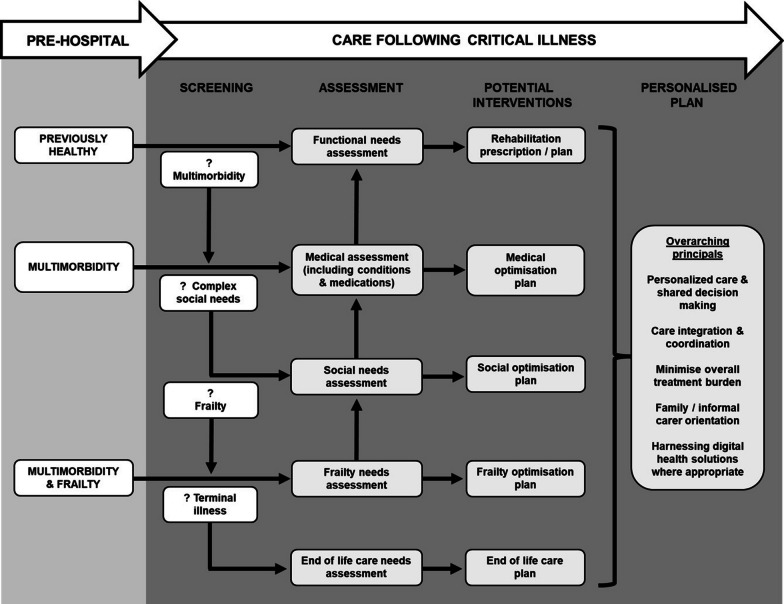
Proposed strategy to adapt care pathways to account for critical illness survivors with multimorbidity and related factors including unmet social needs, frailty, and terminal illness. Each critical illness survivor would be screened for the presence of multimorbidity and related factors. Assessment and optimization would prioritize the lowest relevant level (e.g., end of life care, frailty needs, social needs, medical assessment, and then functional needs), given that each subsequent domain is more challenging to address without addressing the previous unmet needs. The final comprehensive optimisation plan should consider several overarching principals including shared decision making, integration of care, minimized treatment burden, and involvement of family or carers

Existing post-ICU care pathways which usually focus on identification and management of functional impairments could be adapted to account for critical illness survivors with multimorbidity and other important related factors including complex social circumstances and frailty (Fig. 3).

### Identification and optimisation of multimorbidity

Care providers should proactively identify multimorbidity. This includes screening for and optimising important undiagnosed conditions associated with the acute illness that could be contributing to symptoms and increase risk of deterioration, such as undiagnosed COPD, heart failure or obstructive sleep apnoea in survivors of type 2 respiratory failure [50]. Screening for and manAgeing undiagnosed depression and anxiety, which is associated with worse outcomes amongst critical illness survivors [45–47], is likely particularly important, and is one the few multimorbidity interventions which has shown promise in clinical trials [97].

### Identification and optimisation of related factors

Care pathways for critical illness survivors should also proactively identify and address factors closely related to multimorbidity including frailty and complex social circumstances. Models exist in other settings which could provide a framework to simultaneously address a person’s medical, functional, and social problems. Comprehensive Geriatric Assessment (CGA) is a multidimensional holistic assessment of frail older people which simultaneously identifies medical, functional and social problems informing the development of a personalised management plan [101]. CGA could be adapted for critical illness survivors. However, it is time and resource intensive, and evidence indicates it is unlikely to be effective without the leadership of an experienced generalist clinician alongside a complex multidisciplinary team [101]. Any intervention which is introduced should aim to reduce, not increase, treatment burden.

### Personalised care and shared decision making

One of the core features of a multimorbidity model of care is shared decision making and incorporating patient priorities and preferences in management plans [97]. Unlike previous multimorbidity interventions in a stable primary care population which have often failed to demonstrate benefit [97], critical illness survivors with multimorbidity may represent a population whose conditions can be more intuitively prioritised for optimisation, based on their relationship to the acute illness and patient outcomes (Fig. 2).

### Care Integration and coordination

An important distinction between critical illness survivors and other hospitalised patients is additional transitions of care between providers and settings. One of the major challenges for designing care pathways for critical illness survivors with multimorbidity is a lack of clarity on remit and responsibility following ICU discharge, including the roles of ICU, hospital ward and primary care teams. There seems to be consensus that immediate follow-up of functional limitations and rehabilitation following ICU is best provided by ICU teams [91, 95, 102], however these staff may lack the required experience to comprehensively optimise and coordinate care for multiple chronic conditions following ICU discharge [103]. The experience of primary care teams and their long-term relationship with patients makes them well placed to assess multiple new and existing conditions and provide ongoing care. However, the infrequency of critical illness survivors in this setting combined with current resource limitations makes a bespoke intervention for critical illness survivors within this setting challenging. Regardless of their role within any future follow-up intervention, clear communication with the primary care team is vital for long-term continuity of care following hospital discharge [91, 104].

A potential strategy to overcome the lack of clarity on remit and responsibility is incorporation of care coordinators or navigators, who identify unmet needs and integrate with other care providers as required [99, 105, 106]. As well as coordinating care, this model has the potential advantage of being more resource efficient than development of large complex multidisciplinary teams required for models like CGA. However, the comprehensiveness of any assessment will be dependent on the experience level of the care coordinator on the management of multiple common conditions and ability to access support from other professionals when required, which will be highly variable between settings. The Sepsis Transition and Recovery (STAR) post-sepsis transitional care programmeme from the USA utilised a nurse coordinator who liaised with a medical team when required [107]. The STAR programmeme included (1) identification of new physical, mental, and cognitive deficits; (2) review of medications; (3) screening for treatable conditions that commonly lead to poor outcomes; and (4) care coordination. Patients receiving the intervention had significantly lower 30-day mortality or readmission risk compared to controls.

### Treatment burden, self-management support and informal carer orientation

High treatment burden combined with high illness burden, fragile social support and fragmented care significantly reduces the capacity of multimorbid critical illness survivors to self-manage multiple chronic conditions [83, 92, 93]. Interventions to reduce treatment burden and support self-management, including supporting patients to recognise signs of deterioration earlier, is an area which requires further investigation. Self-management interventions will likely need to consider involvement of informal carers and relatives, given their vital role in supporting critical illness survivors following hospital discharge.

### Digital health solutions

The COVID-19 pandemic has led to the rapid adoption of virtual models of care in the post-ICU space [111]. Virtual care has advantages for critical illness survivors and healthcare services including improved efficiency and access, particularly where an ICU covers a large geographic area [112, 113]. For patients with multimorbidity, digital health solutions including virtual care, mobile applications and wearables could also play a key role in supporting self-monitoring and self-management [114]. However, digital care also present challenges, particularly for conditions which require specific clinical assessments or investigations (e.g. blood tests, spirometry or echocardiography). These solutions also have the potential to inhibit access for patients with poor digital health literacy or without the required technology, and has the potential to widen existing health inequalities [111, 115].

Information systems and electronic health records could be harnessed to support optimal patient selection and better integration between care providers, including with hospital specialists and primary care providers. Digitally enhanced decision support tools could be developed to support care providers to deliver evidence-based care for multiple chronic conditions. This could be particularly useful for care providers without generalist experience in chronic disease management to identify unmet needs and gaps in care.

## Conclusion

Most previous research and clinical guidance on provision of post-ICU follow-up has focussed on addressing functional sequelae and symptoms which can be directly attributed to the critical illness and ICU exposure. There is increasing recognition that a person’s pre-existing health status, and in particular the presence of multiple long-term conditions, or multimorbidity, is a key determinant of long-term outcome after ICU. Critical illness survivors with multimorbidity experience unique challenges and likely require a different model of care. Care pathways could be adapted to account for multimorbidity and other important related factors including complex social needs and frailty. However, there are numerous unanswered questions, including whether such a model is feasible within current health systems, which professional groups would be responsible for care delivery and coordination, and whether it improves outcomes for patients.

## Data Availability

Not applicable.
